# Evaluating the effectiveness of a transdiagnostic universal prevention program for both internalizing and externalizing problems in children: two feasibility studies

**DOI:** 10.1186/s13034-022-00445-2

**Published:** 2022-02-03

**Authors:** Kohei Kishida, Noriko Hida, Shin-ichi Ishikawa

**Affiliations:** 1 Organization for Research Initiatives and Development, Doshisha University, 1-3, Tatara Miyakodani, Kyotanabe-shi, Kyoto, Japan; 2 Faculty of Psychology, Doshisha University, 1-3, Tatara Miyakodani, Kyotanabe-shi, Kyoto, Japan

**Keywords:** Universal prevention, Transdiagnostic, Children, Internalizing problems, Externalizing problems

## Abstract

**Background:**

The present study examined the effectiveness of the Universal Unified Prevention Program for Diverse Disorders (Up2-D2) for internalizing and externalizing problems for children aged 9–11 years.

**Methods:**

We used two feasibility studies. The Up2-D2 entailed 12 sessions delivered by teachers; each session was developed based on cognitive-behavioral and positive psychological interventions. In Studies 1 and 2, 58 elementary school children aged 9–11 and 73 elementary school children aged 10–11 attended the Up2-D2. The teachers in Study 1 received 1.5 h of on-site teacher training for learning rationales for interventions, how to run the program, and received ongoing supervision by professionals with mental health expertise. In contrast, the teachers in Study 2 were given self-learning DVD materials in place of on-site training and ongoing supervision.

**Results:**

Mixed models revealed that general difficulties, which is total score of both internalizing and externalizing problems, decreased in Study 1 but not in Study 2. Additional analyses for children with subclinical general difficulties revealed that general difficulties, internalizing problems, and externalizing problems decreased in Study 1, whereas in Study 2, general difficulties and internalizing problems decreased, except for externalizing problems.

**Conclusions:**

These results suggest that on-site teacher training and ongoing supervision are imperative for improving general difficulties in children at a universal level. In addition, universal preventive interventions by classroom teachers without on-site training and continuous supervision might be efficacious for reducing general difficulties and internalizing problems for children with subclinical difficulties.

## Background

Mental health problems affect 10–20% of children and adolescents worldwide [1]. The two most widely studied mental health dimensions are internalizing problems (e.g., anxiety, depression, and emotional symptoms) and externalizing problems (e.g., oppositionality, conduct problems, and attention problems) [2, 3]. In children and adolescents, comorbidities between internalizing and externalizing problems are prevalent [4–6] and measuring a general psychopathology factor that integrates both internalizing and externalizing problems can predict subsequent negative outcomes in adolescents. Such generalized factors offer more information and insight into predicting psychopathology in young people compared to single specific factors that isolate internalizing or externalizing problems [7]. Therefore, interventions that aim to prevent general psychopathology or address general difficulties in children and adolescents are critically needed.

Preventive interventions are a promising avenue for delivering mental health support to large numbers of children and adolescents. Preventive approaches are categorized into three types: indicative, selective, and universal [8]. Indicative interventions target children who already have certain mental health symptoms but do not meet specific diagnostic criteria. Selective interventions target those who exhibit risk factors that can lead to the development of mental disorders. Universal interventions include all children, regardless of the presence of mental health symptoms or risk factors. Although targeted approaches (i.e., regarding indicative and selective prevention) can be more effective than universal approaches in terms of reaching a greater magnitude of effect sizes [9–11], universal prevention offers several advantages: (i) unnecessary screening to detect high-risk factors in children and adolescents; (ii) minimizing stigma from the procedure; and (iii) the inclusion of children and adolescents who may not yet be at risk, but may develop psychopathological symptoms in the future [11, 12]. Several systematic reviews and meta-analyses [9, 11, 13] have revealed that universal preventive interventions are effective for both internalizing and externalizing problems in children and adolescents.

In recent years, transdiagnostic interventions that can treat multiple problems and comorbidities have attracted much attention regarding school-based preventive interventions [14–17]. For example, the EMOTION program [17, 18] and the Super Skills for Life [15, 19] were implemented as transdiagnostic targeted preventive interventions for children and adolescents with emotional symptoms such as anxiety and depression. In addition, the Unified Protocol for Children [14] and the Unified Protocol for Adolescents [16, 20] have also been used as transdiagnostic universal preventive interventions for children and adolescents. However, these transdiagnostic preventive interventions targeted only internalizing problems, such as anxiety and depression, in children and adolescents. Thus, no transdiagnostic preventive intervention has yet been presented that primarily focuses on the integration of internalizing and externalizing problems or general difficulties.

In today’s school settings, it is critical to deliver a prevention program by teachers for large-scale implementation [11]. A meta-analysis [10] that included 43 teacher-delivered controlled trials for elementary school-aged children showed that teacher-delivered preventive interventions, including both universal and targeted prevention methods, were effective for both internalizing (*g* = 0.30; 95%CI, 0.16–0.43) and externalizing problems (*g* = 0.50; 95%CI, 0.35–0.63). Furthermore, teacher training and ongoing supervision from professionals with mental health expertise are of critical importance in enhancing the effectiveness of preventive interventions delivered by teachers. Another meta-analysis [21], including 42 teacher-delivered prevention trials for internalizing problems in adolescents, indicated that preventive interventions, which included two or more days of training before the trials, significantly improved both depression (*g* = − 0.12; 95%CI, − 0.20 to − 0.04) and anxiety (*g* = − 0.14; 95%CI, − 0.26 to − 0.01), while those with one or fewer days of workshops did not significantly improve either depression (*g* = − 0.07; 95%CI, − 0.18 to 0.07) or anxiety (*g* = − 0.07; 95%CI, − 0.19 to 0.07). In addition, regular and in-person supervision significantly improved depression (*g* = − 0.18; 95%CI, − 0.30 to − 0.06), but not anxiety (*g* = − 0.15; 95%CI, − 0.33 to 0.03). Thus, these meta-analyses indicated that universal preventive interventions by teachers are effective for both internalizing and externalizing problems when teacher training and ongoing supervision are provided.

The Universal Unified Prevention Program for Diverse Disorders (Up2-D2 [22]) has been designed as a new transdiagnostic preventive intervention program that can address both internalizing and externalizing problems in children and adolescents. The Up2-D2 entails 12 sessions featuring components from cognitive-behavioral and positive psychological interventions. The program aims to reduce the risk factors that lead to mental health problems and promote protective and resilience factors in children and adolescents. Most importantly, the program was developed based on the principle of user-centered design [23] to enable teachers to implement preventive interventions in school settings. Two feasibility studies were conducted to examine the effectiveness of the Up2-D2. First, a feasibility study for elementary students aged 9 to 12 [24] indicated that 396 children who received the Up2-D2 exhibited increased self-efficacy and social skills and decreased general difficulties. In addition, high-risk children who exhibited apparent autistic traits showed increased self-efficacy and decreased general difficulties after receiving the Up2-D2. Another feasibility study for junior high students aged 12 to 13 [25] indicated that 108 adolescents who received the Up2-D2 reported decreased anxiety levels after receiving the program. Furthermore, a secondary analysis indicated that adolescents with higher symptoms (general difficulties) reported decreased general difficulties, emotional symptoms, and hyperactivity/inattention behavior after attending the universal prevention program. The teachers who were the principal providers in the first study received a one-day teacher training course and were provided with a training DVD to practice with by themselves. Those in the second study also received teacher training (two hours) and on-site supervision by professionals with mental health expertise during implementation. In short, the two feasibility studies provided teachers with on-site teacher training, and then the teachers implemented preventive interventions with their students.

Previous studies [24, 25] have demonstrated that the Up2-D2 can be effective for decreasing general difficulties not only in general samples but also in high-risk or subclinical samples. However, the impact of the types of teacher training and degree of ongoing supervision on the effectiveness of the Up2-D2 has not been investigated. Teacher training and ongoing supervision can be of critical importance in enhancing the effectiveness of preventive interventions delivered by teachers, and further study is needed to determine what degree or kinds of structures of teacher training and ongoing supervision can enhance the effectiveness of the Up2-D2. The present study examined the effectiveness of the Up2-D2 for both internalizing and externalizing problems in children aged 9–11 years, implementing two feasibility studies. One study included on-site teacher training and ongoing supervision, and the other included only self-learning DVD materials in place of training and supervision.

## Method

### Recruitment processes and participants

Study 1 (elementary school A) and Study 2 (elementary school B) had different recruitment processes. First, in February 2018, our research team conducted a workshop that introduced the program to teachers and school personnel in several cities. In Study 1, school personnel participants from School A contacted our research team after the workshop and expressed interest in implementing the program in their school. They wished to integrate this program with School A’s regular curriculum in February 2019. After discussions with the teachers, the stakeholders of School A (i.e., the school principal) consented to the implementation of the Up2-D2 program from September 2020 to February 2021. In Study 2, a member of the local board of education approached our research team in September 2018 to implement the program in all schools throughout the city. Discussions took place with teachers in each school during 2018–2019, and 1 out of the 13 schools in the city agreed to implement all 12 sessions of the Up2-D2 as part of the regular school curriculum in 2020–2021. Finally, the program was implemented in School B from September 2020 to March 2021.

In Study 1, 64 children from School A participated in the Up2-D2 in one 4th grade class and one 5th grade class. Of these, five children were excluded from this study due to incomplete pre-assessment data. Conclusively, data from 58 children (24 boys and 34 girls; 9.91 ± 0.73 years) were used for analyses in Study 1. Seven children (2 boys and 5 girls; 10.43 ± 0.54 years) were defined as subclinical samples, based on a total score of 18 or higher on the Strengths and Difficulties Questionnaire (SDQ [26, 27]). In Study 2, 75 children attended the Up2-D2 from three classes (all being 5^th^ grade) in School B. Of these, two children were excluded due to incomplete pre-assessment data. Conclusively, data from 73 children (39 boys and 34 girls; 10.42 ± 0.50 years) were used for the analyses in Study 2. Ten children (6 boys and 4 girls; 10.70 ± 0.48 years) were defined as subclinical samples, based on the defined criterion mentioned above.

The study was conducted with the approval of the Institutional Review Board of the authors’ university (approval no. 201904 for Study 1 and 202,012 for Study 2). Written informed consent was obtained from the principals of each school. All the children in the classes that participated received the program from their teachers since the program was endorsed as part of the regular school curricula. However, only data obtained through the opt-out consent process from the children were analyzed.

### Intervention

The Up2-D2 [22] is a teacher-delivered cognitive behavioral and positive psychological intervention. The program consists of 12 sessions as universal prevention components in school: psychoeducation about emotion (session 1), behavioral activation (session 2), social skills training (sessions 3 and 4), relaxation (session 5), strength work (session 6), cognitive restructuring (sessions 7 and 8), exposure (sessions 9 and 10), problem-solving (session 11), and review and conclusion (session 12). Each session is designed to last 45–50 min complying with the following flow: a) introduction (i.e., review of the last session and explanation of the goal and purpose of the session); b) learning target skills (i.e., cognitive behavioral or positive psychological skills); c) practicing target skills (i.e., practice of the targeted skills as both individual and group activities); and d) conclusion (i.e., explanation of the homework and summary of the session). Children learn these components along with the worksheets distributed in each session. In addition, teachers provide the Up2-D2 guided by detailed teaching plans that include specific program procedures effectively and effortlessly in school settings.

### Implementation of the Program (Teacher Training and Ongoing Supervision)

The Up2-D2 was implemented by the classroom teachers. In Study 1 (School A), two teachers implemented the program about once every two weeks from September 2020 to February 2021. The teachers from School A received on-site teacher training (1.5 h) to learn rationales for intervention, how to run the program, and ongoing supervision by professionals with mental health expertise during the implementation of the program. During the ongoing supervision, the teachers received on-site supervision by a research member (HN) in session 1. Then, the teachers were able to use ongoing supervision by email from sessions 2 to 12. In Study 2 (School B), three teachers implemented the program about once every two weeks from September 2020 to March 2021. The teachers from School B were given self-learning DVD materials (1.5 h) to learn the rationale and management of the program without on-site teacher training and ongoing supervision.

## Measurements

### Primary outcome

#### Strengths and Difficulties Questionnaire (SDQ)

The SDQs are self-, parent-, and teacher-reported questionnaires that measure children’s emotional/behavioral difficulties and positive attitudes [26]. The Japanese version of the self-reported SDQ [27] was used in this study. The SDQ has five subscales: emotional symptoms, conduct problems, hyperactivity/inattention, peer relationship problems, and prosocial behavior. General difficulties (i.e., total score of all subscales except prosocial behavior), internalizing problems (i.e., total score of emotional symptoms and peer relationship problems), and externalizing problems (i.e., total score of conduct problems and hyperactivity/inattention) were also used in this study. Higher scores indicate higher general difficulties, internalizing problems, and externalizing problems. Based on a previous study of Japanese children [27], children who scored 18 or higher on the total SDQ score were considered as subclinical samples. The internal consistency of the total score of the SDQ was 0.70–0.73 at each point in the two studies, while that for internalizing problems was 0.60–0.68, and for externalizing problems was 0.63–0.73.

### Secondary outcomes

#### General self-efficacy scale for children-revised (GSESC-R)

The GSESC-R is a self-reported scale for general self-efficacy in children [28]. The GSESC-R has two subscales: sensitivity to failure and positive attitude. The total score of the two subscales was used as a measure of self-efficacy. Higher scores indicated higher self-efficacy, sensitivity to failure, and positive attitudes. The internal consistency of the total score of the GSESC-R was 0.79–0.89 at each point in the two studies, while that for sensitivity to failure experience was 0.82–0.92, and for positive attitude was 0.75–0.87.

#### Short version of the Spence Children’s Anxiety Scale (Short CAS)

The Short CAS is a self-reported scale that measures anxiety symptoms in children and adolescents [29, 30]. The reliability and validity of the Short CAS has been confirmed [29]. Higher scores indicate higher anxiety. The internal consistencies of the Short CAS were 0.80–0.87 at each point in the two studies.

#### Depression self-rating scale for children (DSRS-C)

The DSRS-C [31] is a self-reported scale that assesses depressive symptoms in children and adolescents. The reliability and validity of the Japanese version of the DSRS-C have been confirmed [32]. In this study, we used the short version of the DSRS-C developed by Namikawa et al. [33]. Higher scores indicated higher depression. The internal consistencies of the DSRS-C were 0.67–0.75 at each point in the two studies.

#### Anger scale for children and adolescents (ASCA)

The ASCA is a self-reported scale for anger in children and adolescents [34]. The reliability and validity of the ASCA have been confirmed [34]. Higher scores indicate higher anger. The internal consistencies of the ASCA were 0.93–0.97 at each point in the two studies.

### Statistical analyses

All analyses were performed using SPSS 27. First, we examined differences in gender, age, and each outcome at pre-assessment for the total and subclinical samples between Study 1 and Study 2. Second, mixed model analyses were conducted, with time (pre-assessment and post-assessment) as a fixed effect and both individual and class as random effects. Effect sizes (Hedges’ *g*) and 95% confidence intervals (CIs) were calculated. Effect sizes of 0.20, 0.50, and 0.80 were determined to be small, medium, and large, respectively. In addition, based on the results of previous meta-analyses for universal and target preventions for children and adolescents [10, 21], we interpreted small (0.20) or larger effect sizes as meaningful effects for the total samples, and medium (0.50) or higher effect sizes for the subclinical samples.

## Results

### Preliminary analyses

For the total samples, there were no significant differences between Study 1 (School A) and Study 2 (School B) for gender (*χ*^*2*^ = 1.88, *p* = 0.17), general difficulties (*t* = 0.34, *p* = 0.72), internalizing problems (*t* = 0.27, *p* = 0.79), externalizing problems (*t* = 0.46, *p* = 0.65), self-efficacy (*t* = 0.86, *p* = 0.40), sensitivity to failure experience (*t* = 0.33, *p* = 0.75), positive attitude (*t* = 1.11, *p* = 0.27), anxiety (*t* = − 1.18, *p* = 0.24), depression (*t* = 1.20, *p* = 0.23), and anger (*t* = − 0.87, *p* = 0.39). However, the total sample in Study 2 was older than that in Study 1 (*t* = 4.74, *p* < 0.00). For the subclinical samples, there were no significant differences between Study 1 and Study 2 for gender (*χ*^*2*^ = 1.63, *p* = 0.20), age (*t* = 1.01, *p* = 0.29), general difficulties (*t* = 0.15, *p* = 0.88), internalizing problems (*t* = 0.46, *p* = 0.65), externalizing problems (*t* = 0.41, *p* = 0.69), self-efficacy (*t* = 0.23, *p* = 0.82), sensitivity to failure experience (*t* = 0.54, *p* = 0.60), positive attitude (*t* = 0.77, *p* = 0.46), anxiety (*t* = 0.74, *p* = 0.47), depression (*t* = 0.40, *p* = 0.69), and anger (*t* = 1.08, *p* = 0.30).

### Intervention effects for the total samples

For the total sample in Study 1 (School A: *n* = 58), the results revealed that there were significant time effects for general difficulties [*F* (1, 52.78) = 4.88, *p* < 0.05]. In addition, general difficulties decreased with small effect sizes (*g* = − 0.20). However, the results of the other scales of the SDQ and the secondary outcomes were not significant, and effect sizes did not reach the criteria of mean effects. Table 1 shows the results of the mixed model and the effect sizes for the total sample in Study 1. For the total sample in Study 2 (School B: *n* = 73), there were no significant time effects or meaningful effect sizes for all outcomes. Table 2 shows the results of the mixed model and the effect sizes for the total sample in Study 2.

**Table 1:** Mean Scores, Results of the Mixed Model, and Effect Sizes for the Total Sample in Study 1

*N* = 58	Pre			Post			*F*	*g*	95%CI
	*M*	*SD*	*n*	*M*	*SD*	*n*			
General difficulties	11.57	4.89	56	10.58	4.84	52	4.88*	− 0.20	[− 0.58, 0.18]
Internalizing problems	5.75	2.94	57	5.33	3.05	54	1.92	− 0.14	[− 0.51, 0.23]
Externalizing problems	5.86	3.11	57	5.35	2.83	55	3.70	− 0.17	[− 0.54, 0.20]
Self-Efficacy	49.67	9.88	54	50.62	9.99	52	1.24	0.09	[− 0.29, 0.48]
Sensitivity to failure experiences	25.50	5.97	56	26.54	6.51	54	2.15	0.16	[− 0.21, 0.54]
Positive attitude	24.32	5.44	56	24.42	4.91	55	0.12	0.02	[− 0.35, 0.39]
Anxiety	5.60	4.83	58	6.44	5.26	55	2.70	0.16	[− 0.21, 0.53]
Depression	4.44	2.78	57	4.19	2.92	57	0.59	− 0.09	[− 0.45, 0.28]
Anger	2.60	3.96	57	3.02	5.47	57	0.31	0.09	[− 0.28, 0.45]

Note: *p < 0.05

**Table 2:** Mean Scores, Results of the Mixed Model, and Effect Sizes for the Total Sample in Study 2

*N* = 73	Pre			Post			*F*	*g*	95%CI
	*M*	*SD*	*n*	*M*	*SD*	*n*			
General difficulties	11.24	5.13	67	12.03	5.39	63	2.54	0.15	[− 0.19, 0.49]
Internalizing problems	5.60	3.37	68	6.06	3.16	65	2.21	0.14	[− 0.20, 0.48]
Externalizing problems	5.60	3.14	68	6.03	3.56	69	1.12	0.13	[− 0.21, 0.46]
Self-Efficacy	48.20	8.87	66	47.89	11.50	62	0.06	− 0.03	[− 0.38, 0.32]
Sensitivity to failure experiences	25.13	6.68	70	24.51	7.61	67	0.73	− 0.09	[− 0.42, 0.25]
Positive attitude	23.29	4.93	69	23.47	5.27	68	0.32	0.04	[− 0.30, 0.37]
Anxiety	6.66	5.24	73	7.10	4.88	72	0.47	0.09	[− 0.24, 0.41]
Depression	5.08	3.22	72	4.81	2.98	72	0.42	− 0.09	[− 0.42, 0.24]
Anger	3.31	5.07	71	3.64	5.62	73	0.13	0.06	− 0.26, 0.39]

### Intervention effects for the subclinical samples

For the subclinical sample in Study 1 (School A: *n* = 7), although there were no significant time effects for the primary and secondary outcomes, general difficulties, internalizing problems, and externalizing problems decreased with medium effect sizes (*g* = − 0.79, *g* = − 0.52, and *g* = − 0.60, respectively). The other outcomes were not significant and showed more than medium effects, which were interpreted as meaningful effects in this study. Table 3 shows the results of the mixed model and effect sizes for the subclinical sample from School A. For the subclinical sample in Study 2 (School B: *n* = 10), the results revealed that there were significant time effects for depression as a secondary outcome [*F* (1, 9) = 6.45, *p* < 0.05]. General difficulties and internalizing problems, anxiety, and depression decreased with medium effect sizes (*g* = − 0.69, *g* = − 0.55, *g* = − 0.63, and *g* = − 0.63, respectively). However, the results of the other outcomes were not significant and did not show larger than medium effects. Table 4 shows the results of the mixed model and the effect sizes for the subclinical sample from School B.

**Table 3:** Mean Scores, Results of the Mixed Model, and Effect Sizes for the Subclinical Sample in Study 1

*n* = 7	Pre			Post			*F*	*g*	95%CI
	*M*	*SD*	*n*	*M*	*SD*	*n*			
General difficulties	20.29	2.75	7	16.57	5.56	7	4.69	− 0.79	[− 1.87, 0.30]
Internalizing problems	9.29	2.29	7	7.57	3.64	7	4.85	− 0.52	[− 1.59, 0.54]
Externalizing problems	11.00	3.00	7	9.00	3.21	7	3.82	− 0.60	[− 1.67, 0.47]
Self-Efficacy	37.14	6.23	7	39.43	11.00	7	0.43	0.24	[− 0.81, 1.29]
Sensitivity to failure experiences	18.57	4.47	7	20.57	7.39	7	0.87	0.30	[− 0.75, 1.36]
Positive attitude	18.57	5.62	7	18.86	6.07	7	0.03	0.05	[− 1.00, 1.09]
Anxiety	11.00	4.97	7	10.86	6.62	7	0.00	− 0.02	[− 1.07, 1.02]
Depression	8.43	2.82	7	7.86	4.10	7	0.23	− 0.15	[− 1.20, 0.90]
Anger	6.14	6.15	7	7.86	9.72	7	0.14	0.20	[− 0.85, 1.25]

**Table 4:** Mean Scores, Results of the Mixed Model, and Effect Sizes for the Subclinical Sample in Study 2

*n* = 10	Pre			Post			*F*	*g*	95%CI
	*M*	*SD*	*n*	*M*	*SD*	*n*			
General difficulties	20.50	2.88	10	17.89	4.28	9	2.83	− 0.69	[− 1.62, 0.24]
Internalizing problems	10.00	3.62	10	7.89	3.66	9	3.07	− 0.55	[− 1.47, 0.36]
Externalizing problems	10.50	2.07	10	10.00	3.86	10	0.34	− 0.15	[− 1.03, 0.72]
Self-Efficacy	37.90	7.13	10	39.78	12.90	9	0.18	0.17	[− 0.73, 1.08]
Sensitivity to failure experiences	17.20	5.51	10	20.11	8.77	9	1.01	0.38	[− 0.52, 1.29]
Positive attitude	20.70	5.66	10	20.70	6.29	10	0.00	0.00	[− 0.88, 0.88]
Anxiety	12.80	4.92	10	9.00	6.60	10	4.07	− 0.63	[− 1.52, 0.27]
Depression	7.80	3.36	10	5.40	3.86	10	6.45*	− 0.63	[− 1.53, 0.26]
Anger	9.89	7.39	9	8.20	8.38	10	2.74	− 0.20	[− 1.11, 0.70]

## Discussion

The present study examined the effectiveness of the Up2-D2 for children aged 9–11, using two feasibility studies. Results for the total samples revealed that general difficulties decreased in Study 1 (*g* = − 0.20), but not in Study 2 (*g* = 0.15). Additional analyses of the subclinical samples revealed that general difficulties, internalizing problems, and externalizing problems decreased in Study 1 (*g* = − 0.79; *g* = − 0.52; *g* = − 0.60, respectively), whereas general difficulties and internalizing problems decreased in Study 2 (*g* = − 0.69; *g* = − 0.55). These results suggest that on-site teacher training and ongoing supervision are required to gain general difficulties in children as a universal prevention program in schools. Whereas, a universal preventive intervention by classroom teachers without on-site training and continuous supervision might be efficacious for subclinical children to reduce general difficulties and internalizing problems.

The effectiveness of the intervention for general difficulties was seen in the participants of Study 1 (School A), in which children received the Up2-D2 from classroom teachers who received both on-site teacher training and ongoing supervision. This result supports the rationale of the program developed as a transdiagnostic preventive intervention for both internalizing and externalizing problems [22]. However, universal preventive gains were not obtained in Study 2 (School B), where the program training was conducted via DVD and no ongoing supervision was provided. Considering implementations between the two groups, we can discuss several explanations regarding the differences in preventive gains. First, the lack of on-site teacher training and ongoing supervision may prevent teachers from learning and understanding the intervention rationale and how to run the program; as a result, the inherent preventive gains may not be retained. A meta-analysis of teacher-delivered prevention trials for internalizing problems in adolescents [21] showed that two or more days of teacher training or ongoing supervision can increase the effectiveness of teacher-delivered mental health interventions. Although the duration of the on-site teacher training was only 1.5 h in Study 1, it allowed them to ask questions before their implementation due to the provision of ongoing supervision. Moreover, ongoing supervision can continuously provide opportunities for teachers to show tips, solve slips, resolve questions, and troubleshoot all issues before or immediately after each session. Therefore, interactive training and continuous supervision are essential to make the most of universal preventive interventions in schools.

Second, the differences in age between the two samples should be considered. Specifically, children from School A were younger (9.91 ± 0.73 years) than those from School B (10.42 ± 0.50 years). Another meta-analysis [13] indicated age differences regarding the effectiveness of universal preventive interventions for general difficulties compared to control groups. Specifically, the integrated effect sizes were significant in children (*SMD* = − 0.13, 95% CI − 0.24 to − 0.02), but not in adolescents (*SMD* = − 0.04, 95% CI − 0.26 to 0.18). There is no clear evidence what age is appropriate for introducing a universal prevention program in schools and no clear boundary between any grades to produce different effects for emotional problems. Future studies should examine potential moderators, including age and/or grade, in terms of universal preventive programs in schools.

Third, different recruitment processes were applied for the two studies, which could have affected the results. Specifically, School A participated in this project through a bottom-up recruitment process in which school personnel from School A wanted to implement the program in their school and approached our research team. School B participated in this project through a top-down recruitment process where a member of the local board of education requested School B to implement the program and the teacher was willing to do that. Given this difference, teachers from the two schools might differ in their motivations for implementing the program. Although there is no clear evidence that the recruitment process and/or teacher motivation can have an impact on the effectiveness of universal interventions, higher teacher motivation with a bottom-up recruitment process entails a more accurate understanding of the rationale and higher fidelity to the program. Indeed, significant improvement in depression in adolescents compared to control groups was only found for the previous trials delivered by teachers with high and good fidelity, but not for trials with lower fidelity [21]. In the future, the impact of both bottom-up and top-down recruitment processes on teachers’ motivation and fidelity needs to be examined.

For the subclinical children from Schools A and B, the Up2-D2 improved both general difficulties and internalizing problems regardless of training and supervision. This result is consistent with two previous feasibility studies of the Up2-D2 [24, 25]. These studies also revealed that children with a risk factor (high autistic traits) or adolescents with higher symptoms (general difficulties) decreased general difficulties after receiving the universal program. Similarly, several studies reported effectiveness for high-risk groups (high baseline anxiety, depression, hopelessness, or existing parental psychopathology), as well as for universal samples in teacher-delivered prevention programs [21]. Thus, universal prevention programs can be effective for children and adolescents with risk factors or higher mental health symptoms, especially for internalizing problems such as anxiety and depression. However, the results from School A showed that children with general difficulties had decreased externalizing problems, while those from School B without training and supervision did not. In recent years, several universal preventive interventions for anxiety and depression in children and adolescents have been conducted in Japan [35–38]. Therefore, many Japanese teachers are aware of mental health problems related to internalizing problems, such as anxiety and depression, in children and adolescents. The results of School B also showed a decrease in children’s anxiety and depression, and these results supported the hypothesis that teachers are paying more attention to treating anxiety and depression. However, preventive interventions for externalizing problems in children and adolescents are rarely implemented in Japanese school settings. Hence, without teacher training and supervision, it might be difficult for Japanese teachers to recognize and treat externalizing problems in preventive interventions. However, although it is ideal to provide teachers with teacher training and supervision, it is not always possible to do so sufficiently and adequately in real-world school settings because of a lack of resources. Therefore, it is useful in real-world school settings in which a universal program without teacher training and supervision could be effective for decreasing general difficulties and internalizing problems in subclinical samples.

In addition to promoting individual factors (e.g., self-efficacy and cognitive behavioral skills), promoting environmental factors (e.g., peer support and positive classroom environments) are also important and essential for implementing universal prevention interventions in schools [37, 39]. The Up2-D2 includes cognitive behavioral and psychological interventions to develop children’s individual factors. In addition, in this study, the Up2-D2 was conducted in the classroom and included group activities for each session to improve environmental protective/resilience factors. Although the present study did not show effectiveness for self-efficacy, a previous feasibility study of the Up2-D2 indicated that self-efficacy and social skills in children improved after receiving the program [24]. However, target skills in the program (i.e., cognitive behavioral and positive psychological skills) and environmental factors were not measured in either study. Future research needs to measure mediation variables to examine why this program might be effective for generalized difficulties.

There are several limitations to these feasibility studies. First, a control group and follow-up measurements were not used in either study. To examine the effectiveness of the program, it is necessary to include a control group and follow-up in future studies. Second, although this study used only children’s self-reported measures, multiple assessments should be conducted. For example, parent- and teacher-reported measurements of child mental health are required. Third, it is not sure if data were representative for the population of Japanese children in general or not. Future works should collect data such as social issues, family structure, and socio-economic status to examine generalizability of the results of this study. Forth, there was no assessment of the teachers. For example, teacher motivation, teachers’ mental health literacy, and fidelity of intervention delivered by teachers were not measured in the two studies. These outcomes should be measured to examine whether a user-centered design adequately supports the understanding and implementation of the program for teachers. Finally, the data in this study were obtained from only Japanese samples. Although this study showed differences in the effectiveness for internalizing and externalizing problems, we cannot deny a possibility that the results are unique to Japan. Future works should examine whether our findings can be replicated in other countries and cultures.

## Conclusions

Despite several limitations, this study demonstrated that the Up2-D2 with on-site training and ongoing supervision can be effective in reducing general difficulties in general and subclinical samples of children. Even stand-alone implementation can ameliorate general difficulties and internalizing problems in subclinical samples. Although countless preventive interventions for internalizing or externalizing problems have been implemented worldwide [9, 11], there have been very few transdiagnostic preventive interventions that can address general difficulties, or both internalizing and externalizing problems. The Up2-D2 is an intervention that can address both internalizing and externalizing problems, and many children and teachers are expected to receive benefits from the implementation of this program in the future.

## Data Availability

The data that support the findings of this study are available from the corresponding author upon reasonable request.

## Abbreviations

Up2-D2: Universal Unified Prevention Program for Diverse Disorders
SDQ: Strengths and Difficulties Questionnaire
GSESC-R: General Self-Efficacy Scale for Children-Revised
Short CAS: Short version of the Spence Children’s Anxiety Scale
DSRS-C: Depression Self-Rating Scale for Children
ASCA: Anger Scale for Children and Adolescents
SMD: Standardized mean difference
CI: Confidence interval
